# Determinants of childhood vaccination completion at a peri-urban hospital in Kenya, December 2013 -January 2014: a case control study

**DOI:** 10.11604/pamj.2015.20.277.5664

**Published:** 2015-03-20

**Authors:** Okunga Wandera Emmanuel, Amwayi Anyangu Samuel, Kutima Lydia Helen

**Affiliations:** 1 Field Epidemiology and Laboratory Training Program, Ministry of Health, Nairobi, Kenya; 2 Jomo Kenyatta University of Agriculture and Technology, Nairobi, Kenya

**Keywords:** Vaccination, determinants, children, case control, Kenya

## Abstract

**Introduction:**

Vaccine preventable diseases account for about 17% of deaths among children below five years in Kenya. Immunization is one the most cost-effective ways of reducing child mortality and morbidity worldwide. In Kenya, national full vaccination coverage today stands at above 80%. However there continue to be pockets of low full vaccination coverage like the catchment area of Alupe Sub-District Hospital which pose a threat to the rest of the country.

**Methods:**

This was a case-control study at Alupe Sub-District Hospital, Western Kenya. Sixty one (61) cases and 122 controls were sampled from the facility maternal and child health register by systematic random sampling and traced to their households. Cases were defined as children 12-23 months resident in Kenya who received at least one infant vaccine at the facility but were not fully vaccinated at the time of the study, while controls were children 12-23 months who were fully vaccinated by the time of the study. Pretested structured questionnaires were used for data collection. Data entry and analysis was done using Epi-Info 3.5.4 statistical software.

**Results:**

Independent determinants of infant vaccination completion were the child's age < 18 months (AOR 4.2(1.8-9.6), p < 0.01), maternal age < 25 years (AOR 2.5(1.1-5.0), p = 0.03), maternal tetanus toxoid vaccination status < 2 TT doses (AOR 2.5(1.2-5.4), p < 0.02) and late receipt of BCG [AOR 3.2(1.4-7.3), p = 0.005).

**Conclusion:**

Strategies to increase full vaccination should target young mothers especially during antenatal period.

## Introduction

In Africa, the mortality rate in children less than 5 years is 119/1000 live births and in Kenya it is 74/1000 live births [1]. About 17% (1.5 million) of all these deaths under five years are estimated to arise from vaccine preventable diseases. Immunization is one of the most cost effective ways of reducing childhood mortality and morbidity globally. Immunization programs vary worldwide in the diseases they target and strategies applied. In Kenya, the Ministry of Health delivers free immunization through the Division of Vaccines and Immunization. The country targets to achieve at least 90% national vaccination coverage with Pentavalent3, measles and fully immunized child. The country also targets at least 80% immunization coverage with Pentavalent 3, measles and fully vaccinated child in all districts and 90% in all high-risk districts [2]. The country has made great strides in attaining these targets however wide regional and facility level disparities remain which pose a threat to the whole national program. In the former Western province of Kenya, vaccination coverage has been traditionally high with most districts performing at above 80% fully vaccinated child coverage. However, Teso South district performance is very low compared to its neighbouring districts. Fully vaccinated child coverage in 2012 was: Busia 106%, Samia 90%, Butula 86%, Bunyala 88%, Teso North 74% while Teso South was 61%. At Alupe Sub District Hospital which is the biggest facility in Teso South District of Busia County, Western Kenya all immunization indicators remain dismally low. Most children initiate their vaccinations at the facility but completion of the required vaccinations is very low compared to national and county rates. During 2012, the mean annual DPT 1 coverage was 64%, DPT 2 was 53%, DPT 3 was 48%, fully vaccinated child coverage was 43%, measles was 39% and DPT 1/ measles dropout rate was 21% [3]. Clusters of low immunization completion rates have been identified as a major cause of vaccine preventable disease outbreaks in areas with otherwise high immunization coverage. This was seen during polio outbreaks in Oman (1988), Canada (1982), Netherlands (1982), Bulgaria (1991) and Taiwan (1982) [4]. In the United States where endemic measles transmission was eliminated in 2000, outbreaks have continued to occur due to unvaccinated groups [5]. Similarly in Europe where high measles coverage had been attained, concerns about vaccine safety led to under vaccination and subsequent outbreaks [6]. In Africa, measles outbreaks during 2009-2010 were reported to arise due to non-vaccination. Reasons for non-vaccination included vaccine unavailability, infrequent vaccine sessions, exclusion of children > 12 months and unwillingness to receive vaccination by some religious groups in Zimbabwe, Botswana, Malawi and South Africa [7]. Many factors have been associated with low immunization completion rates including immunization system factors, parental attitudes and knowledge, family characteristics and communication and information factors [8–11]. However, the interplay of these factors and their relative significance varies from place to place. This makes it essential for all unique clusters of low immunization to be investigated to identify cluster specific modifiable factors.

## Methods

### Study site

**Figure 1:**
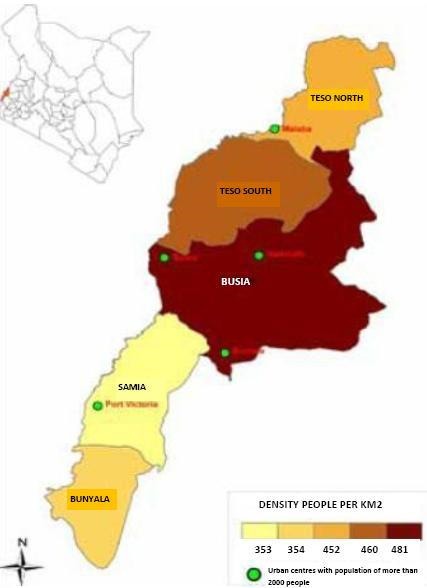
Map of Busia County, Western Kenya showing the constituent sub-counties including Teso South sub-county Map source: Commission on Revenue Allocation-Kenya

The study was conducted at Alupe Sub district hospital in Alupe division of Teso South sub-county, Busia County. The division serves as the main catchment area of Alupe Sub-District Hospital within Kenya. However, the facility also serves the neighbouring Amukura division in Teso North sub county, Busia Sub County in Kenya and the neighbouring Busia district in Uganda. The hospital is a level 4 facility with a catchment population of 40,000. It offers both out-patient and in-patient services. It is currently linked to two community health units. The population served is multi-ethnic though Teso is the predominant ethnic group. Most people are Christians. The majority of the population lives below the national poverty line of KShs 3000 per month. Teso South is a border district with a poverty rate above 60% compared to the national average of 45.9%. National poverty line for Kenya is $ 1.25 per person per day at 2005 purchasing power parity. Literacy rate as per Kenya Integrated Household Budget Survey (2005/06) was 76% with males at 84.2% and females at 68.1%. The predominant religion is Christianity, though Islam is also present as are traditional African religions. The sex ratio is 98 males for 100 females. Children 0-14 years account for 47.5% of the population. Orphan hood was estimated at 10.2% for either one or both parents. 45.9% of the population lives more than 5 kilometers away from the nearest health facility. The average household size is 5.9 members. More than half (57%) of household heads are married in monogamous unions, 23% in polygamous unions, 15% are widowed and 0.3% has never married. Two thirds (66.5%) of households were male led and 33.5% female led. Latrine coverage is above 99% although more than 58.2% of households share latrines. More than 60% of households have access to safe drinking water although about half have to walk more than 30 minutes to access it [12]. Busia County lies in western Kenya along the border with Uganda (Figure 1). It comprises 6 districts (sub-counties). The predominant ethnic communities are Luhya and Teso although there are also a sizeable proportion of other ethnicities including Luo, Kikuyu and Kamba. The main economic activities are subsistence agriculture, fishing and small scale commerce. Poverty is widespread in the county. The district as most of the country uses the six antigen vaccine schedule with the recent introduction of Hepatitis, Haemophilus influenza, Pneumococcal and Rotavirus vaccines. The study assessed the six antigens: BCG and birth polio (at birth-2 weeks), OPV1 and DPT1 (6 weeks), OPV2 and DPT2 (10 weeks), OPV3 and DPT3 (14 weeks) and measles (9 months).

**Study population:** children who received at least one infant vaccine at Alupe SDH during the period 1st September 2011 to 31st August 2012 were studied.

**Study design:** a 1:2 un-matched case-control study design was employed. Two comparison groups, cases and controls, were selected based on a predefined outcome case definition. Two controls were selected for each case to increase the power of the study. Different exposure variables were analyzed against outcome and measures of association were computed to determine significant determinants of full vaccination status in children 12-23 months resident in Kenya that had received at least one infant vaccine at Alupe SDH. The age range was chosen in line with WHO recommendations for immunization coverage surveys for countries where the final primary vaccination was measles at 9 months [13].

**Sample size:** sixty one cases inclusive of an allowance of 10% non-response rate were recruited. These were sampled from 318 defaulters fitting case definition during the period 1^st^ September 2011 to 31^st^ August 2012. One hundred and twenty two controls inclusive of 10% allowance for non-response rate were recruited from 1518 fully vaccinated children in the MCH register for the period 1^st^ September 2011 to 31^st^ August 2012. Two controls were recruited for every case. Sample size was estimated by Fleiss formula with continuity correction using OpenEpi software. A prevalence of FIC among mothers who had completed primary education of 87% and among those who had not completed of 67% was applied [1]. Other assumptions were: 95% confidence level, power 80% and least extreme odds ratio to be detected 0.03. The sample was increased by 10% to accommodate for non-response. This brought cases to 61 and controls to 122.

**Sampling frame:** all children 12 - 23 months resident in Kenya who received at least one infant vaccine at Alupe SDH between 1^st^ September 2012 and 30^th^ August 2013 and were documented in the MCH register at the health facility were listed then stratified into cases and controls based on a predetermined case definition. Three hundred eighteen (318) cases and 1464 controls were extracted from the MCH register. A sample of 61 cases and 122 controls were enrolled by systematic random sampling. A random number between 1 and 318 was generated by OpenEpi software and assigned as the first case, and then every fifth case was enrolled till 61 cases were enrolled. The same procedure as for cases was used. A random number between 1 and 1518 was generated and every twelfth control was enrolled till 122 controls were enrolled.

**Case definition:** a child aged 12 - 23 months resident in Teso South, Kenya who has received at least one infant vaccine Alupe SDH but has not completed all vaccinations up to first dose of measles.

**Case finding:** all children fitting case definition extracted from MCH register were traced to their households using household registers maintained by community health workers (CHWs) and assisted by the village elders/chiefs. Vaccination status was confirmed by card for 59 children and by history and BCG scar for 2 children. Those not found were systematically replaced until we reached the anticipated sample size. Those found but were fully vaccinated were excluded from the study.

**Control finding:** controls were sampled from the MCH register and traced to their households. Controls were children aged 12-23 months who started their primary infant vaccination schedule at Alupe SDH and were now fully vaccinated. One hundred and twenty two controls were recruited and traced to their households. Vaccination status was confirmed by card for 119 children and by history and BCG scar for 3 children. We systematically replaced those not found until the anticipated sample size was attained.

**Inclusion criteria:** children, who received at least one infant vaccination at Alupe SDH, are aged 12 -23 months, were resident in Teso South, Kenya and parents or guardians consented to inclusion in study.

**Exclusion criteria:** children who were resident in Uganda were excluded.

**Fieldwork and data collection:** community entry was through the area chief, community health extension worker and CHWs acted as guides to homesteads of the study participants. They also introduced the researcher to the community members through informal meetings. Voluntary informed consent from mothers or care-givers of children using detailed informed consent forms in common English with verbal translation to Kiswahili and/or local languages where necessary was obtained. An interpreter, who was a CHW from the locality, accompanied the researcher to assist in case of language barriers. Data was collected through face to face interviews with mothers or guardians using structured interviewer administered standard WHO EPI survey questionnaires merged with a poverty index tool (Mark Schreiner, Microfinance Risk Management, L.L.C., 2441 Tracy Avenue, Kansas City, MO 64108, U.S.A., mark@microfinance.com) with minimal adjustments for local context. Mothers of children not fully vaccinated were given appropriate health education and advised on completion of the vaccination schedule where appropriate. Mothers who were willing to have their children vaccinated were linked to the facility and followed up by CHWs.

**Data management:** a pilot study involving 10% of the main study sample size was conducted. Responses not anticipated in initial data collection tool were identified and final coding of the questionnaire was done. Practical field problems related to language barrier were noted and appropriate translators identified. The relatively high number of anticipated study participants resident in Uganda was unexpected. However, sampling was continued till target sample size was found in the main study. Data was entered into an Epi Info 3.5.4 make-view screen installed on a password protected computer with appropriate back up. All cover pages of questionnaires with personal identifiers were destroyed immediately after data entry and cleaning while the main questionnaires were retained by the researcher. Data analysis was done using the same software.

### Variables

**Input variables:** demographic indicators selected for the study and elicited through interviewer administered questionnaires were: child's age, gender and birth order, place of residence, paternal age and maternal age. Socioeconomic factors selected and elicited through interviewer administered questionnaires were parental income, education level and religion. Children were further grouped into two groups: 12-17 months and 18-23 months to represent the younger and older halves of the study children's age group. Mothers were grouped into two also: (=) 25 years since half of the study mothers were aged below 25 years. Healthcare access indicators chosen and elicited through interviewer administered questionnaires were: distance from health facility, migration in the preceding two years, antenatal clinic attendance (at least once during index pregnancy) confirmed by ANC card, receipt of age appropriate birth polio and receipt of age appropriate BCG. Health system factors selected and also elicited through interviewer administered questionnaires were facility utilization indicators: place of delivery, receipt of at least 2 doses of neonatal tetanus by mother confirmed by ANC card (143/183 study mothers/care-givers had ANC cards) and age appropriate receipt of late vaccinations (OPV 3, DPT 3 and measles). Reasons for non-vaccination given by mothers/care-givers as well as main sources of maternal information, education and communication on immunization services were elicited.

**Output variable:** full immunization status at 12-23 months of age was the output/dependent variable. This variable is the standard WHO measure of full infant vaccination.

**Data analysis:** frequency tables were drawn using Epi Info data analysis program. Percentages were calculated and compared. Two by two tables were generated with calculated odds ratios, their 95% confidence intervals and tests of significance (p-values) to measure association between known determinants of immunization and full immunization status. Multivariate analysis by backwards elimination method was done. Variables with a p-value (< = ) 0.1 from bivariate analysis were entered into a model and by sequentially eliminating those with p-values (>) 0.05, starting with the one with the highest p-value, the best fit model was reached. Dependent variables entered were: age-group of the child, age-group of the father, age-group of the mother, age-appropriate BCG, source of maternal IEC being a CHW, source of maternal IEC being a friend, age-appropriate OPV3 and receipt of (= >) 2 doses of tetanus toxoid. This was done to identify interactions among factors identified from bivariate analysis.

**Ethical considerations:** the requisite consent was sought from the medical superintendent to conduct the study. Voluntary informed consent was sought from each child's mother or guardian interviewed. The consent was written, detailed and in simple language without medical jargon. It was in English and Kiswahili. Verbal translation into local language was done where necessary by a trained study interpreter. Ethical approval to conduct the study was obtained from the Kenyatta National Hospital Ethical and Research Board. No personal information was disclosed to third parties during conduct of the study. All study participants’ parents or guardians were given health education on vaccine preventable diseases and immunization. Confidentiality of all participant information was ensured as per international guidelines on protection of human research participants.

## Results

### Study participants

One hundred and eighty three (183) study participants aged 12-23 months who had received at least one infant vaccination at Alupe Sub-District Hospital and were resident in Kenya were enrolled. Of these 61 were cases and 122 were controls. All study participants were from Alupe division. Majority of children were from two locations: Angorom 82(45%) and Amongura 75(41%).

### Demographic factors

Cases were more likely to be younger than 18 months [OR 4.0(2.0-8.2),<0.01’> p < 0.01], likely to have to have younger fathers [OR 0.5(0.3-1.0)p = 0.04] and younger mothers [OR 0.4(0.2-0.8)p = 0.01]. However they were similar with respect to card availability [OR 0.7(0.1-4.6)], sex [OR 1.3(0.7-2.5)p = 0.43] and birth order [OR 0.7(0.4-1.3), p = 0.27].

### Household and socioeconomic characteristics

Cases were similar to controls in respect of all household and socioeconomic determinants of immunization reported in other studies (Table 1, Table 2, Table 3).

**Table 1:** Household characteristics of study participants

Household characteristic	Cases(n)	%	Controls(n)	%	OR, 95% C.I.	p-value
Rural	33	57	68	56		
Urban	28	43	54	44	1.0(0.6-1.4)	0.88
**Distance from water source**						
0-30 minutes	59	97	120	98		
>30 minutes	2	3	2	2	0.5(0.1-3.6)	0.6
**No. of habitable rooms in house**						
One	41	67	85	70		
Two or more	20	33	37	30	0.9(0.5-1.7)	0.87
**Floor type**						
Wood, soil, other	53	87	107	88		
Cement or tiles	8	13	15	12	0.9(0.4-2.3)	0.87
**Lighting fuel**						
Firewood, Paraffin	58	95	118	97		
Electricity	3	5	4	3	0.7(0.1-3.0)	0.69
**Ownership of mosquito nets**						
None	4	7	7	6		
One or more	57	93	115	94	1.2(0.3-4.1)	1
**Access to media**						
**Radio**						
Yes	51	84	106	87		
No	10	16	16	13	0.8(0.3-1.8)	0.65
**Television**						
Yes	3	5	4	3		
No	58	95	118	97	1.5(0.3-7.0)	0.69
**Internet**						
Yes	18	30	25	20		
No	43	70	97	80	1.6(0.8-3.3)	0.2

**Table 2:** Paternal socioeconomic characteristics of study participants

Paternal characteristic	Cases(n)	%	Controls(n)	%	OR, 95% C.I.	p-value
**Income(Ksh)**						
<3000	33	54	54	45		
3000 +	28	46	66	55	1.4(0.8-2.7)	0.27
**Occupation**						
None	2	3	1	1		
Peasant agriculture, Small business, other	58	96	119	99	4.1(0.4-46.6)	0.26
**Highest education level attained**						
Has not completed primary school	20	33	45	38		
Completed at-least primary school	40	67	75	62	1.2(0.6-2.3)	0.7
**Religion**						
Christian	59	98	114	95		
Other	1	2	6	5	3.1(0.4-26.4)	0.43
**Marital status**						
Monogamous	45	75	103	85		
Polygamous, Other	15	25	17	15	0.5(0.2-1.1)	0.1
**Has received information on immunization**						
Yes	60	100	117	98		
No	0	0	3	2	3.1(0.2-62.4)	0.51

**Table 3:** Maternal socioeconomic characteristics of study participants

Maternal characteristic	Cases(n)	%	Controls(n)	%	OR, 95% C.I.	p-value
**Income(Ksh)**						
<3000	47	78	91	75		
3000 +	13	22	31	25	1.2(0.6-2.6)	0.71
**Occupation**						
None	12	20	21	17		
Peasant agriculture, Small business, Other	48	80	101	84	1.2(0.5-2.6)	0.68
**Highest education level attained**						
Has not completed primary school	28	46	63	52		
Completed at-least primary school	33	54	59	48	0.8(0.4-1.5)	0.53
**Religion**						
Christian	59	98	119	97		
Other	1	2	3	3	1.5(0.2-14.6)	1
**Marital status**						
Monogamous	45	75	102	84		
Other	15	25	20	16	0.6(0.3-1.3)	0.23
**Has received information on immunization**						
Yes	59	97	120	98		
No	2	3	2	2	0.5(0.1-3.6)	0.6

### Factors related to access to immunization services

Delayed receipt of birth OPV and BCG were the only significant access factors associated with full immunization coverage (Table 4). Distance from health facility was not a significant determinant of full vaccination.

**Table 4:** Health facility access factors

Determinant	Cases	(%)	Controls	(%)	OR,95%CI	p-value
**Migration in the last 5 years**						
Yes	10	16	15	12		
No	51	84	107	88	1.4(0.6-2.7)	0.5
**Distance from health facility**						
<1km	8	13	27	25		
= > 1km	53	87	95	75	0.5(0.2-1.3)	0.17
**Age-appropriate birth OPV**						
No	10	16	11	9		
Yes	32	84	108	91	3.1(1.2-7.9)	0.02
**Age-appropriate BCG**						
No	30	53	33	28		
Yes	27	47	86	72	2.9(1.5-5.6)	<0.01

### Health system factors

The statistically significant health system factors/indicators were delayed receipt of OPV3 (OR 3.0(1.5-6.3) p = 0.02) and failure by the mother to receive (= > ) 2 doses of antenatal tetanus toxoid (OR 3.2(1.6-6.5) < 0.01’> p < 0.01) (Table 5). There were no statistically significant differences between cases and controls in respect of sources of maternal IEC from bivariate analysis. However, CHWs (OR 0.4(0.2-1.1), p = 0.05) and friend (OR 0.6(0.2-1.1), p = 0.06) were included in the multivariate analysis model. Cases and controls were similar in respect of getting their immunization IEC from radio (p = 0.52), television (OR 1.2(0.3-5.2)p = 0.53), healthcare workers (OR 0.6(0.2-2.4)> p = 0.35) and neighbours (OR 1.0(0.5-2.0) p = 0.52). The main reasons for non-vaccination given by case-mothers/care-givers (n = 61) were vaccine stock-outs (33%), mother too busy(8%), unaware of time of vaccination(8%), place of vaccination too far(7%), unaware of need for vaccination(7%), child ill brought but not vaccinated(5%), time of vaccination inconvenient(4%) and other not specified(18%).

**Table 5:** Analysis of health system determinants

Health system indicator	Cases (n)	%	Controls (n)	%	Odds Ratio, 95% Confidential Interval	p-value
Age-appropriate OPV3						
No	21	40	21	18		
Yes	32	60	98	82	3.0(1.5-6.3)	0.002
Age-appropriate DPT3						
No	18	35	32	27		
Yes	34	65	87	73	1.4(0.7-2.9)	0.2
Age-appropriate Measles						
No	8	15	14	12		
Yes	45	85	105	88	1.3(0.5-3.4)	0.35
Mother attended ANC clinic						
Yes	60	98	116	97	0.5(0.1-4.4)	0.5
Place of delivery						
Health facility	31	51	60	51		
Traditional birth attendant	30	49	62	49	1.1(0.6-2.0)	0.5
Mother has received (= >) 2 doses of tetanus toxoid						
No	30	49	24	27		
Yes	25	45	64	73	3.2(1.6-6.5)	<0.01

### Multivariate analysis of risk factors (Unconditional Logistic Regression)

Variables with p = < 0.1 from bivariate analysis included in the model were child age group, maternal age group, paternal marital status, timely birth OPV, timely BCG, timely OPV3, (= > ) 2 doses of maternal tetanus toxoid, receipt of immunization IEC from CHWs and receipt of immunization IEC from friends. Only four factors remained statistically significant after unconditional logistic regression as shown in (Table 6).

**Table 6:** Multivariate analysis of risk factors with p = < 0.1 from bivariate analysis

Characteristic	Unadjusted OR, 95% Confidence limit	p-value	Adjusted OR, 95% Confidence interval	p-value
**Age group of child(months)**				
<18				
18-23	4.0 (2.0-8.2)	<0.01	4.2 (1.8-9.6)	<0.01
**Age group of mother**				
15-25				
>25	0.4(0.2-0.8)	<0.01	2.5(1.1-5.0)	0.03
**Age-appropriate BCG**				
No	2.9(1.5-5.6)	0.001	3.2 (1.4-7.3)	0.005
**Child protected against neonatal tetanus (Maternal TT = > 2)**				
No	3.2(1.6-6.5)	<0.01	2.5 (1.2-5.4)	<0.02

## Discussion

Younger children were less likely to be fully immunized at 12 months probably reflecting fewer chances for immunization. This could also arise due to a recent period of poor immunization services such as healthcare workers strikes as occurred in Kenya in November-December 2013 [14, 15]. These findings are consistent with studies in most sub-Saharan countries [16]. Children of younger mothers were more likely to be incompletely immunized when confounding was controlled for. This is consistent with many studies conducted in similar settings. Paternal age was not a significant determinant of full immunization. In this study gender was not found to be associated with incomplete immunization as found in India [17–19]. Similarly birth order which has been found to be associated with incomplete immunization in Nigeria and India was not a statistically significant determinant of incomplete immunization. Place of residence (urban/rural) did not influence completion rates. Paternal and maternal income, education and religion were not significantly associated with full immunization status. Higher maternal education attainment has been associated with improved immunization completion rates in many similar settings. Physical access to immunization was not a barrier to immunization completion. Distance from health facilities and migration have been associated with low full immunization status [9, 20–23]. In this study area, most participants lived more than one kilometre away from health facilities but this did not affect completion rates. This could be attributed to better means of transportation particularly the presence of motorcycle riders. Few participants had migrated out of the area but even so this did not affect immunization completion rates. Non-physical barriers indicated by low coverage with age-appropriate birth vaccines (OPV and BCG) were noted to exist. Half of the study participants were delivered by TBAs. This could have contributed to the low coverage by age-appropriate vaccines. Non-availability of vaccines when children presented themselves for birth vaccines may also have contributed to low coverage with age-appropriate vaccines. This is both an access as well as health system determinant of full immunization.

Health system utilization was a significant determinant of full immunization as indicated by age-appropriate coverage with OPV 3, DPT 3 and measles. Owino and colleaguesworking in a slum area in Nairobi, Kenya found that health system utilization remained low in spite of excellent access to immunization services [20]. Delivery at a health facility and attending ANC at least once during the index pregnancy was not significantly associated with higher completion rates however attending ANC more than twice as indicated by receipt of at least 2 tetanus toxoid doses was significantly associated with full immunization. This suggests that the more contact with the health system during ANC period the more likely one is to complete infant vaccinations. This is consistent with findings by Tadesse and Belachew in Ethiopia [8, 24], Hu et al in Chinese immigrants [23] and Rahman and Obaida-Nasrin in Bangladesh [22]. Neonatal tetanus protection is an indicator of good ANC attendance and general acceptance of antenatal services. This was also found in Mali [25]. Strategies to improve full immunization coverage should target increasing ANC attendance. The most common reason for children not being vaccinated was vaccine stock outs. Weaknesses in EPI services have long been recognized as deterrents to immunization completion [26, 27]. Evaluation of the source of information on immunization revealed that CHWs and friends may be an important way of reaching mothers as compared to the more traditional modes such as the provincial administration, healthcare workers and radio.

### Study Limitations

Children resident in Uganda were not included in the study due to administrative reasons. This could have introduced information bias. Case findings efforts of the present study traced 38/61(62%) from the original sample and the control finding efforts traced 85/122(70%). Through key informant interviews with the community leaders, the missed children were noted to reside on the Ugandan side of the border. It was noted also that the parents of these children register them as Kenyan residents because the health facility charges non-Kenyan residents more to access charged services. This unexpectedly high number of non-residents may have biased our study. This is a special population that needs study.

## Conclusion

The main determinants of full immunization were child's age (12-17 months), maternal age (= < 25 years), timely receipt of birth OPV and receipt of at least 2 doses of neonatal tetanus toxoid by the mother. Physical distance, socioeconomic and household factors were not significant determinants of full vaccination as reported in many studies in similar settings. Strategies to increase full immunization should aim at increasing the frequency of ANC attendance to at least twice per pregnancy, ensuring comprehensive IEC to the whole community with special focus to younger mothers, early initiation of the vaccination schedule and improving health system performance. CHWs and peer education through friends may be an important addition to traditional modes of immunization IEC for mothers.
